# Editorial: Mindful eating and mindfulness-based practices for healthier eating

**DOI:** 10.3389/fpsyg.2026.1796326

**Published:** 2026-02-18

**Authors:** Michail Mantzios, Jean Kristeller

**Affiliations:** 1 Department of Psychology, School of Health and Life Sciences, Birmingham City University, Birmingham, United Kingdom; 2 Department of Psychology, Indiana State University, Terre Haute, IN, United States

**Keywords:** emotional processes, health related outcomes, mindful eating, mindful eating behavior, mindfulness, motivation, self-regulation

Mindfulness-based practices, including mindful eating, have received increasing attention as constructs that enable and support healthier eating behaviors, psychological wellbeing, and foster broader health outcomes across both clinical and non-clinical populations (e.g., Kristeller et al., 2014; Muñoz-Mireles et al., 2023). The present Research Topic brings together empirical, experimental, and review-based contributions that collectively add to the understanding of how mindfulness-based constructs and interventions may shape eating self-regulation, emotional processes, motivation, and health-related outcomes.

Several contributions in this set of Research Topic papers provide construct-level considerations, whereby the assumption of mindfulness and mindful eating subcomponents contributing uniformly to behavior was challenged. Yang et al. examined individuals with polycystic ovary syndrome and infertility, and their findings showed that mindfulness sub-constructs such as *acting with awareness* may indirectly reduce emotional eating through lower depressive symptoms. Meanwhile, a mindfulness sub-construct, that is*, observation*, was associated with increased emotional eating. Such results suggest that heightened awareness alone may be insufficient, without appropriate contextual framing or skill development, or in this context, the higher “observation” may be related to awareness (and worry) of increases in emotional eating for some people. Concerns about mindfulness constructs are further echoed in work addressing measurement and operationalization (Grossman, 2019). Hussain et al., across three studies, explored relationships between mindful eating behavior, decision-making for mindful eating, and descriptions of self-compassion. Their findings indicated that sensory attention and non-judgmental awareness are the components most consistently linked to self-compassion, another mindfulness-based construct, indicating how measurements in mindful eating may provide conflicting findings, while associations with body mass index remain elusive in current literature. These findings clarify the importance of including measures of eating behavior and overeating, which mindfulness-based programs are highly effective in treating (Kristeller and Jordan, 2018; Mason et al., 2016). Together with the findings of Yang et al., these two articles reinforce the need for conceptual clarity and careful selection of mindfulness-based measurements and constructs when interpreting outcomes and designing interventions. While findings are largely positive, the field is challenged by variability in definitions and measurement tools, intervention formats, proposed mechanisms of change, and effectiveness across populations and contexts (e.g., Grossman, 2019; Mantzios, 2023, 2025), concerns that need to be considered in the schema of strictly relying on cross-sectional data.

Extending beyond measurement problems and implications for practice, Moore et al. situated mindfulness within broader patterns of motive-driven eating. They found that lower dispositional mindfulness was uniquely associated with coping-, reward-, social-, and conformity-driven over-consumption of highly palatable foods. Significantly, coping- and reward-driven eating were more prevalent among individuals reporting mental health disorders, positioning mindfulness as a potential factor that links and buffers eating behavior for those with greater psychological vulnerability. Similarly, Ghabashi, using a broad measure of eating self-regulation that included healthy nutritional elements, found that eating fruit daily and regular breakfasts were related to less general anxiety among a large Saudi Arabian sample (93% women), and to higher self-esteem and satisfaction with daily life.

While some of the literature centers on individual health and focuses on internal benefits of mindfulness in this Topic, other contributions fostered external benefits by expanding the scope of mindfulness and mindful eating into fields of sustainability and dietary transitions. Giannou and Mantzios examined mindful eating, gratitude, and motivations to reduce food waste, and showed that both mindful eating and gratitude are positively associated with moral and financial motivations for waste avoidance. Despite mindful eating not moderating the gratitude–food waste relationship, non-judgmental awareness did, highlighting the role of self-regulation of attention, with eating being instrumental to support sustainable food practices. A similar expansion beyond individual behavior was evident in work on dietary change. Winkelmair and Jansen, in a randomized trial, evaluated the effects of two distinct mindfulness interventions and a stress-reduction program on affective evaluations of vegetarian foods. Improvements in explicit attitudes across groups, alongside the influence of social and personal norms on goal intentions, illustrated how internal psychological processes interact with social context to shape dietary transitions. Together, these contributions harness support for broader pro-environmental behaviors and sustainable food systems, enhancing the impact beyond individualistic benefits into socio-ecological perceptions and responsibilities. Importantly, transcending a narrower focus on individual health, and potentially self-centric health goals, by embracing broader sustainability and value-driven action, may provide a more robust and sustainable approach to enhancing individual health.

A key focus across contributions was the recognition and value of how mindfulness influences eating behavior, as already noted. Chu et al. open their paper with an overview of the negative effects of chronic dieting. Using a creative daily assessment approach, they show that body image mediates the inverse relationship between state mindfulness and overly restrictive eating. These findings emphasize the role of embodiment within mindfulness, suggesting that changes in how individuals relate to their bodies may be fundamental to more adaptive eating regulation. Exploring brain activity, Logemann-Molnár et al. show that dispositional mindfulness is associated with alterations in the balance between voluntary and stimulus-driven attention, independent of food-reward context and frontal brain asymmetry. Dispositional mindfulness appears to support attentional flexibility, providing additional support on how dispositional mindfulness may operate within eating-related decision making, protecting people from excessive focus on weight, while heightening appreciation of the quality of foods eaten.

Finally, the translational potential of mindfulness-based approaches was synthesized in a narrative review by Shao et al. that examined mindfulness-based interventions for adolescent obesity. Their review indicated that mindfulness can positively influence both physiological and psychological indicators during a critical developmental period, situating mindfulness-based practices as promising components of early prevention strategies, and aligning with findings in other research within this Topic Issue, with adult populations.

Collectively, the research contributions in this Research Topic issue demonstrate that mindfulness and mindful eating exert their effects through multiple, interacting pathways that encompass self-perception, attention, emotional regulation, motivation, and social context. Mechanistic insights, construct-level considerations, mental health relevance, and broader societal outcomes support a more mechanism- and context-sensitive application of mindfulness-based and mindful eating practices for future research and interventions in the field.

## References

[B1] Grossman P. (2019). On the porosity of subject and object in ‘mindfulness' scientific study: challenges to ‘scientific' construction, operationalization and measurement of mindfulness. Curr. Opin. Psychol. 28, 102–107. doi: 10.1016/j.copsyc.2018.11.008 30583202

[B2] Kristeller J. Wolever R. Q. Sheets V. (2014). Mindfulness-based eating awareness treatment (MB-EAT) for binge eating disorder: a randomized clinical trial. Mindfulness 4, 282–297. doi: 10.1007/s12671-012-0179-1

[B3] Kristeller J. L. Jordan K. D. (2018). Mindful eating: connecting with the wise self, the spiritual self. Front. Psychol. 9:1271. doi: 10.3389/fpsyg.2018.01271 30154740 PMC6102380

[B4] Mantzios M. (2023). Mindful eating: a conceptual critical review of the literature, measurement and intervention development. Nutr. Health 29, 435–441. doi: 10.1177/02601060231153427 36703297

[B5] Mantzios M. (2025). “Eating isn't just about paying attention—it's about the self-regulation of sensory attention while eating!”: exploring mindful eating by examining sensory attention and non-judgmental awareness in the context of eating cessation. Nutr. Health 31, 1237–1242. doi: 10.1177/02601060241289508 39397561 PMC12423464

[B6] Mason A. E. Epel E. S. Kristeller J. Moran P. Dallman M. Lustig R. H. . (2016). Effects of a mindfulness-based intervention on mindful eating, sweets consumption, and fasting glucose levels in obese adults: data from the SHINE randomized controlled trial. J. Behav. Med. 39, 201–213. doi: 10.1007/s10865-015-9692-8 26563148 PMC4801689

[B7] Muñoz-Mireles G. Mantzios M. Schellinger J. N. Messiah S. E. Marroquín E. (2023). Mindful eating as a tool for diabetes prevention and management: a review of potential mechanisms of action. Mindfulness 14, 2831–2847. doi: 10.1007/s12671-023-02236-y

